# Editorial: Design strategies and equipment requirements for efficient process development and robust manufacturing of cell therapies

**DOI:** 10.3389/fbioe.2026.1875958

**Published:** 2026-07-06

**Authors:** Christoph Herwig, Volker Huppert, Ralf Pörtner

**Affiliations:** 1 Körber Pharma Software, Wien, Austria; 2 Glycostem Therapeutics B.V., Oss, Netherlands; 3 Act for Hope Foundation, London, United Kingdom; 4 Hamburg University of Technology, Hamburg, Germany

**Keywords:** ATMP, bioreactor, cell assays, cell manufacturing, cell therapy, PAT (process analytical technologies), process development

## Introduction

Cell-based therapies represent a paradigm shift in medicine, offering transformative options for oncological, immunological, and degenerative diseases refractory to conventional treatment. As the pipeline of cell therapy products advances towards commercialization, the field confronts a defining challenge: the manufacturing process has an impact on Critical Quality Attributes, and therefore patient safety and commercial viability, as much as the biology itself. Living cells as therapeutics impose demands on process design and equipment that differ fundamentally from classical biologics manufacturing—variable starting materials, mechanical sensitivity, multistep processing, and stringent regulatory requirements demand closed, reproducible, and scalable production platforms.

This Research Topic was conceived to address these demands, spanning: bioprocess development for T cells, NK cells, mesenchymal stromal cells (MSCs), and pluripotent stem cells; equipment characterization for bioreactors and retention devices; process analytical technology (PAT) and digital tools; viral vector and product analytics; and the quality management frameworks governing decentralized and point-of-care manufacturing. The fourteen contributions collected here map the engineering and technological landscape across these themes.

## Automation, PAT, and digital infrastructure

The transformation toward knowledge-driven manufacturing requires innovation across three interdependent layers. Szarzynski et al. frame this as “CGT 4.0”: closed automated unit operations, miniaturised real-time biosensors for metabolic monitoring, and digital twin models for predictive process control. The authors, representing the PAT4CGT consortium, argue that hybrid mechanistic-data-driven modelling is the most practical near-term route to prescriptive control, and critique conventional bioreactor sensor arrays as wholly insufficient for characterizing complex CGT batches.

Reliable PAT requires aseptic, small-volume access to process samples. Chan et al. present Auto-CeSS, an automated sampling system withdrawing ≥30 μL at ≥15-min intervals from a 2 mL perfusion microbioreactor (Breez) and an 8 mL gas-permeable well-plate (G-Rex). Over 12-day T-cell expansion, Auto-CeSS yielded glucose, lactate, glutamine, and glutamate profiles statistically indistinguishable from manual sampling, with sterility confirmed under USP-compliant testing even outside biosafety cabinet conditions. For adherent culture in large-format stacked vessels, Mason et al. deploy a cloud-integrated software platform using a random forest pixel classifier to automate hiPSC confluency estimation at 99.4% pixel accuracy, streaming results with spatiotemporal heatmaps to an interactive GMP-ready dashboard - addressing a biomass monitoring gap with no previous industrial solution.

Analytical quality control of gene-modified cell therapies depends on accurate lentiviral vector (LVV) potency measurement. Farcas et al. demonstrate that standard 96-well plate titre assays systematically underestimate LVV potency because the 1–3 mm fluid overlay exceeds the ∼0.6 mm effective diffusion range of LVV particles at 37 °C. A PDMS-on-COC microfluidic device with a 0.2 mm channel depth overcomes this mass transport limitation, yielding ∼2-fold higher transduction efficiency across MOIs of 0.five to four, inter-assay CV of 3.9%, linearity R^2^ = 0.9798, a 4-h incubation option, and sensitivity down to MOI 0.0625 - validated by qPCR and undetectable by conventional plates. This establishes a device-based analytical standard with direct implications for QC reproducibility across CGT manufacturing sites.

## Bioprocess development for T-Cell, NK-Cell, and MSC platforms

Perfusion strategies are central to achieving the cell densities and dose outputs required for allogeneic therapy manufacturing. Springuel et al. show, through a DoE study in stirred-tank bioreactors with serum-free/xeno-free medium, that adaptive ATF perfusion achieves 4.5-fold higher CAR-T yields, 50% faster time to clinical dose, and 11% less medium use compared to fixed-rate perfusion, while preserving phenotype and cytolytic function across donors. In a companion manufacturing-scale study, Springuel et al. translate these findings to a 2 L bioreactor yielding 113 CAR-T doses per batch, with multifrequency dielectric spectroscopy monitoring viable cell density in real time (R^2^ = 0.98), an automated Ksep 400 centrifugal harvester for closed product recovery, and the Ambr 250 validated as a predictive scale-down model. For NK cell therapy, Kok et al. evaluate the CliniMACS Prodigy across 36 manufacturing runs, demonstrating consistent, GMP-compliant CD34^+^ enrichment and NK cell concentration from umbilical cord blood in a single, fully closed instrument—confirming that platform devices consolidating multiple unit operations can decisively reduce variability and manual handling in allogeneic cell manufacturing.

MSC expansion presents distinct challenges due to anchorage dependency, shear sensitivity, and donor variability. Herbst et al. systematically review 76 bioreactor MSC expansion studies, identifying agitation rate, dissolved oxygen, pH, glucose, and inoculation density as key CPPs and concluding that the near-universal reliance on endpoint offline measurements—rather than continuous metabolite monitoring—is the principal barrier to knowledge-driven MSC process design. Schneider et al. address the equipment dimension directly, comparing ATF and TFDF (tangential flow depth filtration) cell retention systems for hMSC perfusion on microcarriers in 1.8 L bioreactors. ATF enabled microcarrier aggregate size control and automated multi-step harvest washing; TFDF unexpectedly promoted spheroid formation—a structurally distinct product morphology with different CQA implications. The choice between retention devices thus shapes not only process efficiency but fundamental product characteristics.

## Cell isolation, analytics, and pluripotent stem cell scale-up

Quality control and cell characterisation tools must match the speed and sensitivity demands of manufacturing timelines. Nikoniuk et al. survey microfluidic platforms - droplet systems, surface capture, impedance cytometry, and microwell devices - for CAR-T QC, concluding that despite strong single-cell resolution capabilities, the absence of GMP-compatible integration with bioprocessing equipment is the primary barrier to inline PAT adoption, and propose a roadmap toward closed-loop analytical integration. Cheng et al. present a low-cost, PMMA-based centrifugal lab-on-a-disk (LoaD) device using CD44 magnetic bead labelling and dual-stage rotation to capture 85%–99% of breast cancer cells from heterogeneous populations in under 2 hours - without external pumps or cleanroom infrastructure - with potential applications in liquid biopsy and autologous therapy starting material enrichment.

Human iPSCs as feedstocks for downstream differentiated products require culture systems that protect fragile aggregates from hydrodynamic shear. Tokura et al. describe a 10 L closed bioreactor using an inert plastic fluid with intermittent agitation, achieving a mean of 1.09 × 10^10^ hiPSCs per batch across three independent runs with maintained pluripotency markers (OCT4, SOX2, NANOG, SSEA-4) and normal karyotype - demonstrating that unconventional fluid mechanics approaches can resolve the fundamental engineering tension between agitation-driven nutrient homogeneity and mechanical protection of stem cell aggregates at scale.

## Manufacturing environment and quality management

Process innovation must operate within manufacturing environments and quality systems that ensure regulatory compliance and patient access at scale. Chiu et al. consider the case for pharmaceutical-grade isolators as GMP-compliant infrastructure for point-of-care (POC) cell therapy and extracellular vesicle production in hospital settings, providing Class A sterility assurance without dedicated cleanroom construction - a viable strategy for reducing logistical barriers, shortening apheresis-to-infusion timelines, and improving access to autologous therapies. Von der Leyen et al. provide the regulatory architecture for this distributed model, proposing a QMS framework centred on a central Control Site holding the manufacturing licence, housing the Qualified Person, and overseeing a network of regional Point Of Care sites. The framework maps the regulatory positions of MHRA (modular manufacturing licence, enacted January 2025), FDA (FRAME programme), and EMA (EudraLex Volume 4), and defines the comparability protocols, technology transfer workflows, and electronic QMS tools required to demonstrate product consistency across geographically distributed manufacturing.

## Conclusion

The fourteen contributions assembled here advance the engineering and scientific foundations of cell therapy manufacturing across a broad spectrum of modalities, platforms, and process stages. The common thread is an aspiration toward manufacturing systems that are closed, automated, data-rich, and quality-by-design governed - systems that can consistently deliver high-quality products and ultimately realise the clinical promise of cell therapy for patients at scale.

